# Comparison of subject-to-template registration schemes using CT and MR radiotherapy images with brain lesions

**DOI:** 10.3389/fnins.2026.1858490

**Published:** 2026-07-28

**Authors:** Anna Bangiri, Stefanie Thust, Paul S. Morgan, Stefan Pszczolkowski

**Affiliations:** 1 School of Medicine, University of Nottingham, Nottingham, United Kingdom; 2 Nottingham University Hospitals NHS Trust, Nottingham, United Kingdom; 3 Nottingham NIHR Biomedical Research Centre, University of Nottingham, Nottingham, United Kingdom; 4 Department of Translational Neuroscience and Stroke, UCL Queen Square Institute of Neurology, London, United Kingdom

**Keywords:** CT, MRI, radiosurgery, radiotherapy, registration, voxel-based analysis

## Abstract

**Introduction:**

Voxel-based analyses have been used more widely in radiotherapy in recent years. The purpose of this study is to compare eight different methods of registering images on to a template space for such analyses. A novel way, using both CT and MR data, is proposed.

**Methods:**

CT and MR brain images from 85 participants in the CoDe-B-Rad study (NCT06466720) were registered on an age-specific template. The registration schemes used included sCT-Template (linear and non-linear); MR-Template (linear, non-linear, masked, and enantiomorphic); and Dual-Template (linear and non-linear). The registrations were compared qualitatively and quantitatively against the template MR images with scores ranging from 1 (lowest) to 5 (highest) and quantitatively (via Jaccard, ASD, and HD95).

**Results:**

Qualitatively, the best registration scheme was the Dual-Template-non-linear registration, with 60 participants scoring above 4. The second best was the MR-Template-enantiomorphic, with 53 participants scoring above 4. Quantitatively, the Dual-Template-non-linear method outperformed on the mean (±SD) for the ASD and HD95, with 0.918 (±0.1774) and 2.965 (±0.5392) respectively. For ASD the difference compared to other methods was significant (*p* = 0.03). The MR-Template-masked outperformed on the Jaccard mean (±SD), median (IQR), and ASD, achieving values of 0.567 (±0.0557), 0.555 (0.055), and 0.808 (0.162) respectively. The Dual-Template-non-linear and MR-Template-masked and non-linear had the same result for the median HD95: 2.639. The performance of all linear schemes was inadequate both quantitatively and qualitatively. All non-linear registration schemes had issues with distortions of tissue and landmarks, however, for the Dual-Template scheme these were minimal.

**Conclusion:**

The Dual-Template-non-linear registration scheme is a new way of registering lesioned brain images for use with voxel-wise techniques in radiotherapy, which utilises both CT and MR image data. The scheme provides fidelity of the underlying soft tissue as well as the surrounding skull, minimising anatomical and dosimetric distortions.

## 1 Introduction

In brain radiotherapy, intrasubject rigid image registrations between Magnetic Resonance (MR) and Computed Tomography (CT) images are mainly used. Rigid image registrations involve linear transformations (translations and rotations) and mutual information is used as a metric for the CT to MR registrations (Viola, 1995; Collignon et al., 1995). Linear registrations are most commonly applied, with non-linear registrations reserved for when anatomical changes are expected, such as in the liver, pancreas, or post-surgery (Oh and Kim, 2017; Sarrut, 2006; Crum et al., 2004).

In recent years, voxel-wise analyses, such as Voxel-based Lesion symptom mapping (VLSM) or voxel-based morphometry (VBM), have been more widely used. They utilize the normal tissue response to radiation instead of the more traditional dose-volume histogram (DVH) approach (Thames et al., 2004; Bates et al., 2003; Ashburner and Friston, 2000; Mechelli et al., 2005; Ebert et al., 2021; Sosa-Marrero et al., 2023). With these methods, inter-participant image registrations are required to bring all participant data onto a common space or template (Monti et al., 2018; Monti et al., 2020; Mcwilliam et al., 2023; Baldo et al., 2022). Registering MR images from different subjects to a common template space can be problematic, and even more so when the images are lesioned. These lesions can significantly affect registrations and cause the introduction of additional artefacts regardless of their location (Jühling et al., 2024).

Previous studies have compared various ways of registering lesioned MRI images, such as using masking or enantiomorphic techniques (Monti et al., 2018; Monti et al., 2020; Jühling et al., 2024; Palma et al., 2020; Wilson et al., 2024).

The purpose of this study was to compare and appraise eight different methods of registering lesioned brain MR images on to a common template. Additionally, we propose and test a new approach of linear and non-linear registrations using both CT and MR images simultaneously, which we term dual registration. To the best of our knowledge, dual registration has not been previously proposed in the context of registration for group studies using radiotherapy planning data. We aim to identify which registration process achieves the best results for participants using quantitative and qualitative assessments.

## 2 Methods

### 2.1 Participant population

All 91 participants from the CoDe-B-Rad study (NCT06466720, IRAS 340408, London Stanmore REC Reference 24/PR/0426) were included from both the prospective and retrospective cohorts. The inclusion and exclusion criteria for the cohorts are detailed in the Supplementary materials. They had either brain metastases or meningiomas and were treated with Stereotactic Radiosurgery at the Nottingham University Hospitals NHS Trust.

As part of their clinical treatment, all participants had CT scans (Canon Aquillion Large Bore Exceed scanners), and T1-weighted MR images acquired on a variety of 1.5 T and 3 T scanners from all major vendors. As patients had their MRI scans in different hospitals across the East Midlands region, radiotherapy specific immobilisation devices were not used during scanning.

The corresponding clinical radiotherapy dose distributions from all participants were used (Monaco Treatment Planning System, v.5.11.01, Elekta AB, Stockholm, Sweden). All dose plans were calculated with 1% uncertainty on a 1 cm x1 cm x 1 cm dose grid. The planning technique used was single-isocentre VMAT and the patients were treated on a 6 FFF VERSA HD® linear accelerator (Elekta AB, Stockholm, Sweden).

### 2.2 Template

The template used was the age-specific template from Rorden et al. (2012), which was constructed from a population with a similar age distribution to CoDe-B-Rad. The authors developed a T1-weighted image and a CT version. However, these were constructed from different populations and do not match each other spatially. For this reason, a synthetic CT (sCT) version was generated from the T1-weighted template image using MVision AI, Helsinki, Finland.

### 2.3 Image processing

The clinical intra-participant CT-to-MR registrations were completed in the Raystation Treatment Planning System, v 6.0 (Raysearch Laboratories, Stockholm, Sweden). These were used to transform the CT images into the MR space. Registrations of MR and transformed CT images to template space were carried out with the Medical Image Registration Toolkit (MIRTK) (BioMedIA/MIRTK, 2001) using the following energy functions for optimisation:


$$E_{\mathrm{CT}}(A_{\mathrm{CT}},I_{\mathrm{CT}})=\mathrm{CC}(A_{\mathrm{CT}},T(I_{\mathrm{CT}}))+\lambda \cdot \mathrm{BE}(T)$$ (1)

$$E_{\mathrm{MR}}(A_{\mathrm{MR}},I_{\mathrm{MR}})=\mathrm{CC}(A_{\mathrm{MR}},T(I_{\mathrm{MR}}))+\lambda \cdot \mathrm{BE}(T)$$ (2)

$$\begin{array}{c} E_{\text{dual}}(A_{\mathrm{CT}},A_{\mathrm{MR}},I_{\mathrm{CT}},I_{\mathrm{MR}})=0.5\cdot \mathrm{CC}(A_{\mathrm{CT}},T(I_{\mathrm{CT}}))\\ +0.5\cdot \mathrm{CC}(A_{\mathrm{MR}},T(I_{\mathrm{MR}}))\\ +\lambda \cdot \mathrm{BE}(T) \end{array}$$ (3)

**Figure 1:**
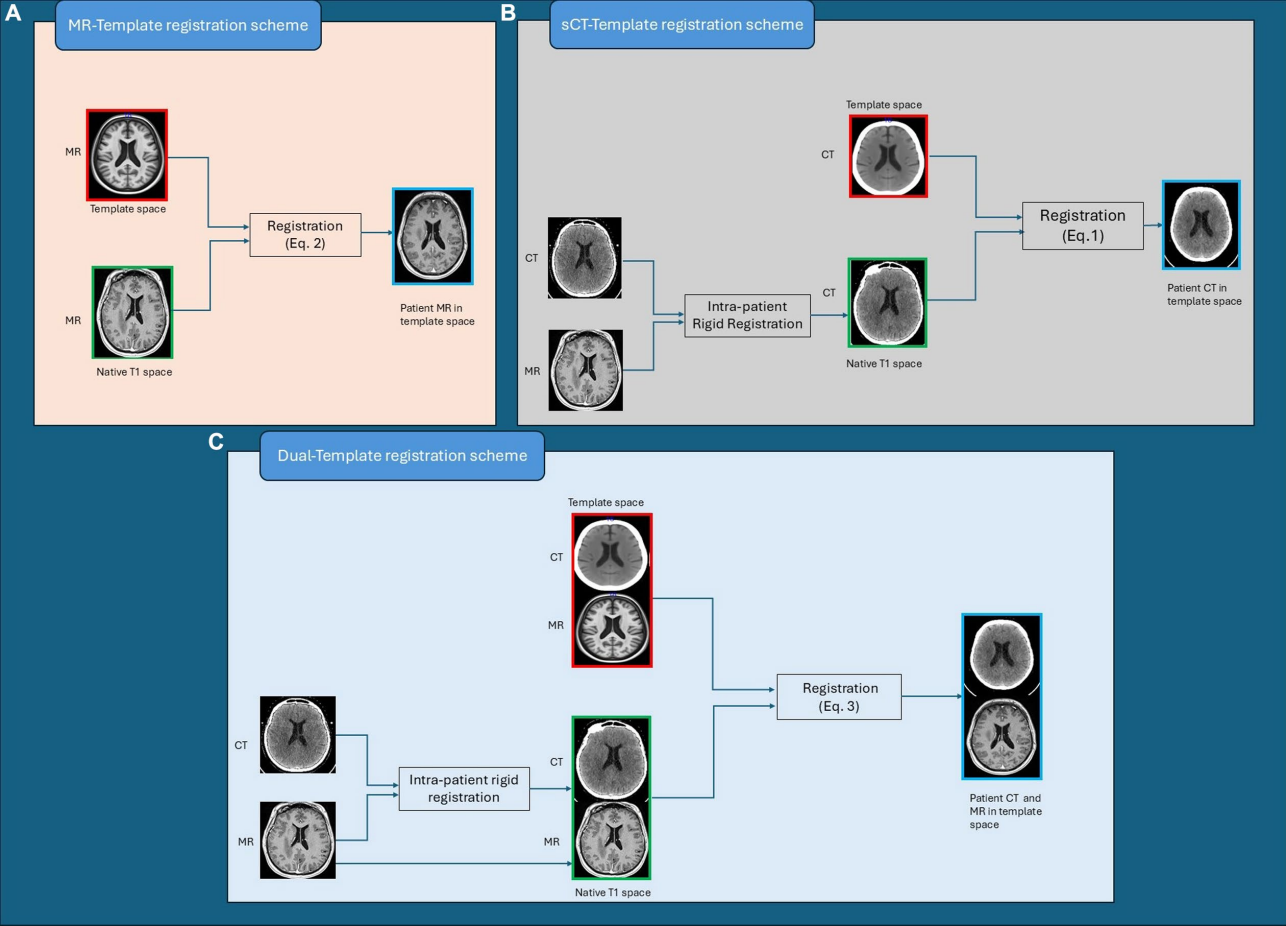
Graphical depiction of the pipelines to bring the patient images onto the template space using the MR-Template linear, non-linear, masked, and enantiomorphic schemes **(A)**, the sCT-Template linear and non-linear schemes **(B)**, and the Dual-Template linear and non-linear schemes **(C)**.

Here, $A_{m}$ and $I_{m}$ correspond to the template and participant image of modality m , respectively. T is the transformation (linear or non-linear) being optimised. The similarity term CC is a local normalised cross-correlation measure previously described in the work by Avants et al. (2008) and BE is the thin-plate bending regularisation energy of non-linear transformations (as it is always zero for linear transformations) (Wahba, 1990). Finally, the weighting parameter λ controls the amount of regularisation and was set to 0.001. Eight different registration schemes were used to bring the participant images onto the template space. A summary of the schemes is provided in Table 1. In Figure 1 the pipelines for the sCT-Template schemes (A), MR-Template schemes (B), and Dual-Template schemes (C) can be seen.

**Table 1:** Description of each registration scheme.

Registration scheme	Description	Optimisation
‘sCT-Template-linear’	Linear registration of CT image in MR space to CT template	Equation 1
‘sCT-Template-non-linear’	Linear then non-linear registration of CT image in MR space to CT template	Equation 1
‘MR-Template-linear’	Linear registration of MR image to MR template	Equation 2
‘MR-Template-non-linear’	Linear then non-linear registration of MR image to MR template	Equation 2
‘MR-Template-masked’	Linear then non-linear registration of masked MR image to MR template	Equation 2
‘MR-Template-enantiomorphic’	Linear then non-linear registration of enantiomorphic MR image to MR template	Equation 2
‘Dual-Template-linear’	Linear registration of CT image in MR space and MR image to CT template and MR template simultaneously	Equation 3
‘Dual-Template-non-linear’	Linear then non-linear registration of CT image in MR space and MR image to CT template and MR template simultaneously	Equation 3

Participant MR images were brain extracted using HD-BET (Isensee et al., 2019), then bias-field corrected using N4 (Tustison et al., 2010), and finally segmented into 138 cortical and subcortical structures using MALP-EM (Ledig et al., 2015). These structures were subsequently combined into a set of 28 structures, prior to any of the registrations taking place (see Supplementary material for details).

We also defined two additional structures for quantitative analyses, yielding a final set of 30 structures. The first structure corresponds to a “cerebral exterior” label defined as all voxels within the HD-BET brain mask that were excluded by MALP-EM (i.e., were given a label of zero). The second structure corresponds to “bone” and was defined as all voxels having Hounsfield Units of between 500 and 2000 on the corresponding CT image in MR space, restricted to within a mask obtained by performing 15 iterations of morphological dilation on the HD-BET brain mask to exclude the patient immobilisation device visible in CT. The age-specific MR template was segmented using MALP-EM, and the resulting labels combined into the final 30 structures in the same way as for participant brain images and using the synthetic CT template to define bone. The final segmentations were brought onto the template space by applying the resulting transformation of each of the 8 registration schemes.

The affine registrations of each scheme were performed according to the definition of that scheme. For example, the MR-Template-masked was initialised with an affine registration using a masked version of the image. More details along with the precise MIRTK parameter settings can be found here https://doi.org/10.17605/OSF.IO/5UPQ8, where we have shared the masking and registration scripts along with the sCT images. All non-linear registrations were constrained in the target space by use of a brain mask, with the similarity metric computed only within that mask.

The radiotherapy dose distributions were used to create both the masked and enantiomorphic images. The dose prescriptions in our SRS participant cohort varied between 12 Gy^1^ and 22 Gy in 1 fraction (ICRU, 2016). For brain metastases, multiple lesions can be treated in one plan, with varying prescriptions included.

Some participants in our cohort received varying doses for different lesions in the same radiotherapy treatment within the same treatment plan. Fifteen Gy was an overestimate of the lesion volume in the majority of the cases, but for the participants with multiple prescriptions in one radiotherapy plan only a common mask with one level would be appropriate. There were three participants with meningiomas with prescriptions less than 15 Gy (two were prescribed 12 Gy and one 14 Gy). The remaining 78 had prescriptions between 15 and 22 Gy. As such, 15 Gy was used as the threshold dose of interest to create the masks.

To create the enantiomorphic image, the intensities within the lesion mask are replaced by the equivalent healthy tissue intensities from the contralateral side of the brain.

### 2.4 Analysis

#### 2.4.1 Qualitative evaluation of registrations

Each transformed participant MRI scan was overlaid with the template MR and reviewed using ITK-SNAP version 3.8.0 (Yushkevich et al., 2006). A radiotherapy Clinical Scientist (AB), with over 10 years of experience in reviewing registrations for SRS treatments, carried out the qualitative assessment. The entirety of size, shape, and overlay of the ventricles, brainstem, corpus callosum, whole brain, and skull were considered for registration quality. Additionally, the position of the midline and the position and length of the straight sinus were examined. Each registration was scored on a scale from 1 to 5. One represented the lowest alignment quality, with gross mismatches in the majority of the structures mentioned above. A score of two was given when the gross mismatches did not cover the entirety of the structures. A score of three showed better agreement with the template but with some obvious distortions (e.g., dips in the skull or the corpus callosum or an over-extension of the straight sinus) in one or two structures. A score of four showed overall good agreement in most structures, with only small deviations in structure positioning. Finally, a score of 5 signified the best possible score, where all structures along with the shape and size of the brain and skull were fully aligned.

Intra-rater reliability was assessed using a two-way mixed-effects, absolute agreement, single-rater Intraclass correlation coefficient (ICC (3,1)) across the different methods for 20 patients, after a washout period of 3 months.

#### 2.4.2 Quantitative evaluation of registrations

For each participant, each of the 30 structure labels was compared with the corresponding one on the template using the Jaccard coefficient, defined as follows (Jaccard, 1912):


$$\mathrm{JAC}=\frac{|X\cap Y|}{|X\cup Y|}$$

Where X and Y are the set of voxels of a segmented structure in the transformed MRI of the participant and the MR template, respectively.

As additional quantitative measures, we used the average surface distance (ASD) and Hausdorff distance (HD). The ASD corresponds to the average distance between the closest points of the boundaries of the two structures. The HD corresponds to the maximum of these distances. However, to avoid biases due to outliers, we used the 95th percentile of these distances, commonly known as HD95. The mean (±SD) and median (IQR) scores for Jaccard, ASD, and HD95 across the 30 structures was calculated for each patient and each scheme. The mean (±SD) and median (IQR) scores for each scheme across all patients were also calculated.

Statistical analyses using IBM SPSS Statistics (version 29.0.1.0) were performed. The medians for Jaccard, ASD, and HD95 were tested for normality using the Shapiro–Wilk test along with visual inspection of normal Q-Q plots. The different registration methods demonstrated a mixed distribution. The Friedman test was used to evaluate the differences between the eight registration methods, with post-hoc pairwise comparisons, where statistical significance was demonstrated. To control for Type I error rate, the Dunn-Bonferroni correction was applied to all post-hoc pairwise significance levels. The level of statistical significance was set at 0.05 for all tests.

The means for each registration method was tested for normality using the Shapiro–Wilk test along with visual inspection of normal Q-Q plots. This was done for Jaccard, ASD, and HD95. The different registration methods demonstrated a mixed distribution. However, due to the large sample size (*N* = 86), a parametric one-way repeated measures ANOVA was used to compare the means for the eight different registration methods. The Greenhouse–Geisser estimates were used to correct the degrees of freedom where sphericity was violated. Post-hoc pairwise comparisons were performed with a Bonferroni correction to control the family wise error rate. The level of statistical significance was set at 0.05 for all tests.

A short timing study was carried out to assess whether the Dual-Template significantly adds to the computation time. A total of 10 patients were timed and the average time per patient was compared across Dual-Template-non-linear, sCT-non-linear, and MR-Template-non-linear. Finally, we assessed the behaviour of the non-linear transformations in the lesion area and elsewhere by evaluating the local Jacobian determinants.

## 3 Results

From the 91 participants recruited, one participant was recruited in error and five were recruited both in the prospective and retrospective cohorts. The latter were excluded to avoid duplication and, as such, 85 participants were included in the final analysis. Thirty-four patients had meningiomas (40%) and 51 (60%) had brain metastases. The per-patient total tumour volumes ranged from 0.5 cc to 52.8 cc. The participants were predominantly female 59% and of a White ethnic background (93%).

### 3.1 Qualitative evaluation of registrations

**Figure 2:**
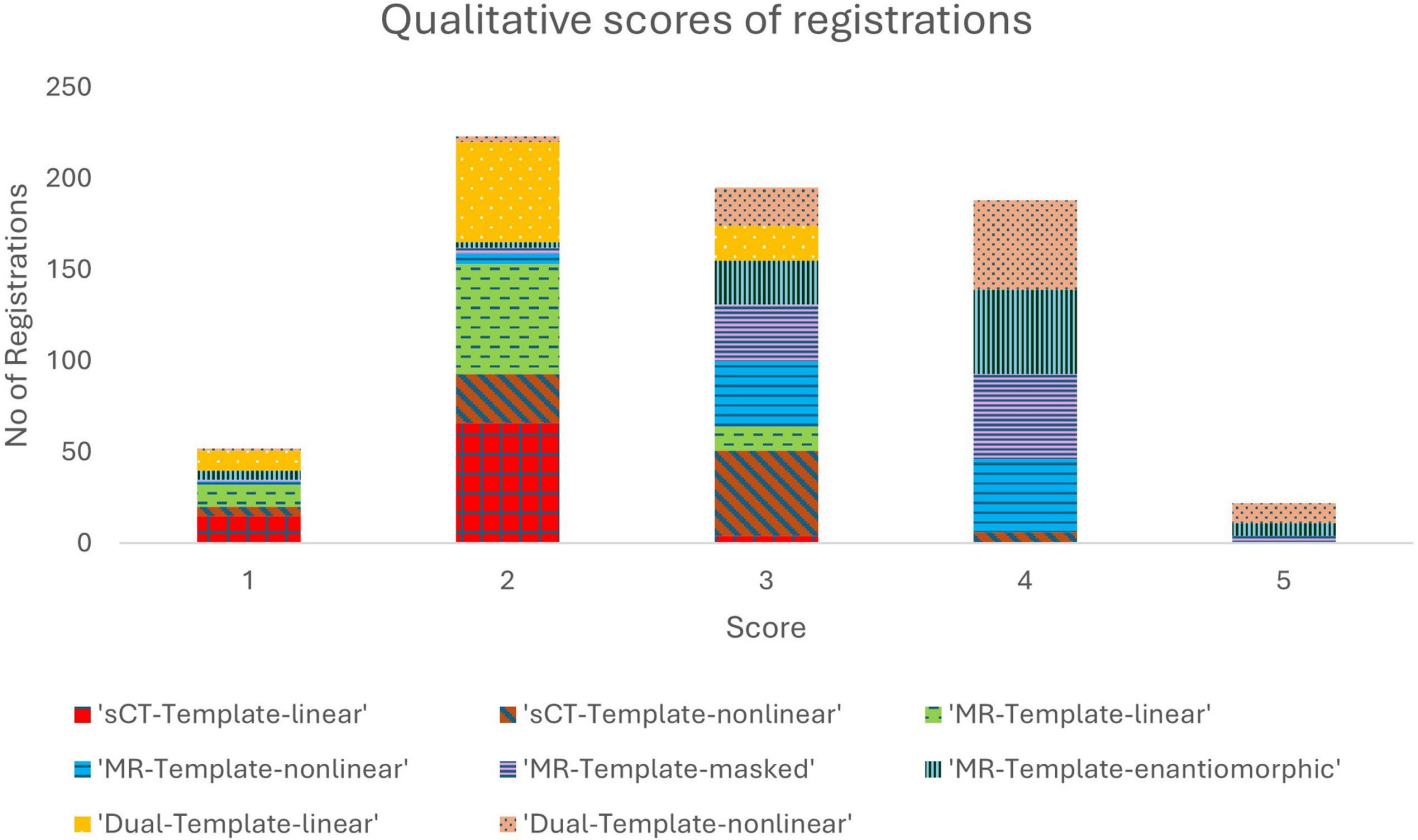
The qualitative rankings for all participant registrations across each scheme.

The Dual-Template-non-linear registration had the largest number of participants, with a score of 4 or 5 with n = 60. The second and third largest number of participants with such scores corresponded to the MR-Template enantiomorphic and MR-Template-masked schemes, with 53 and 50 participants, respectively. The linear registrations on all schemes had no participants evaluated with a score higher than 3. The graph with the entire distribution of participant scorings for all registrations is shown in Figure 2.

**Figure 3:**
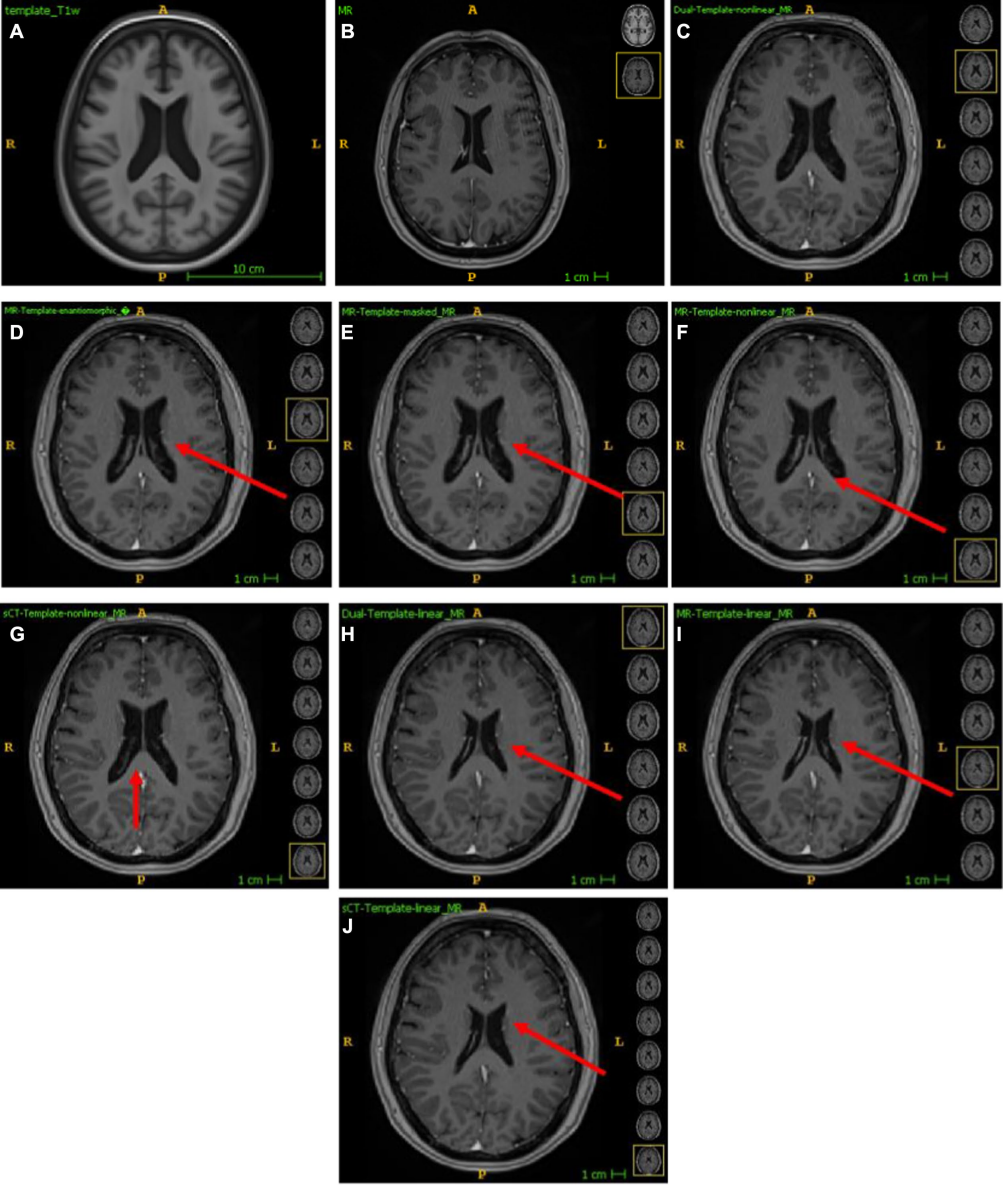
Comparison of axial slices of the MR images registered on to the template space **(A)**, the native space **(B)** using the Dual-Template-non-linear **(C)**, MR-Template-enantiomorphic **(D)**, MR-Template-masked **(E)**, MR-Template-non-linear **(F)**, sCT-Template-non-linear **(G)**, Dual-Template-linear **(H)**, MR-Template-linear **(I)**, and sCT-Template-linear schemes **(J)**. The images are shown from highest to lowest score, with the Dual-Template-non-linear scoring a 5 and the sCT-Template-linear scoring a 2. The red arrows point to distortions in the shape and location of the ventricles.

**Figure 4:**
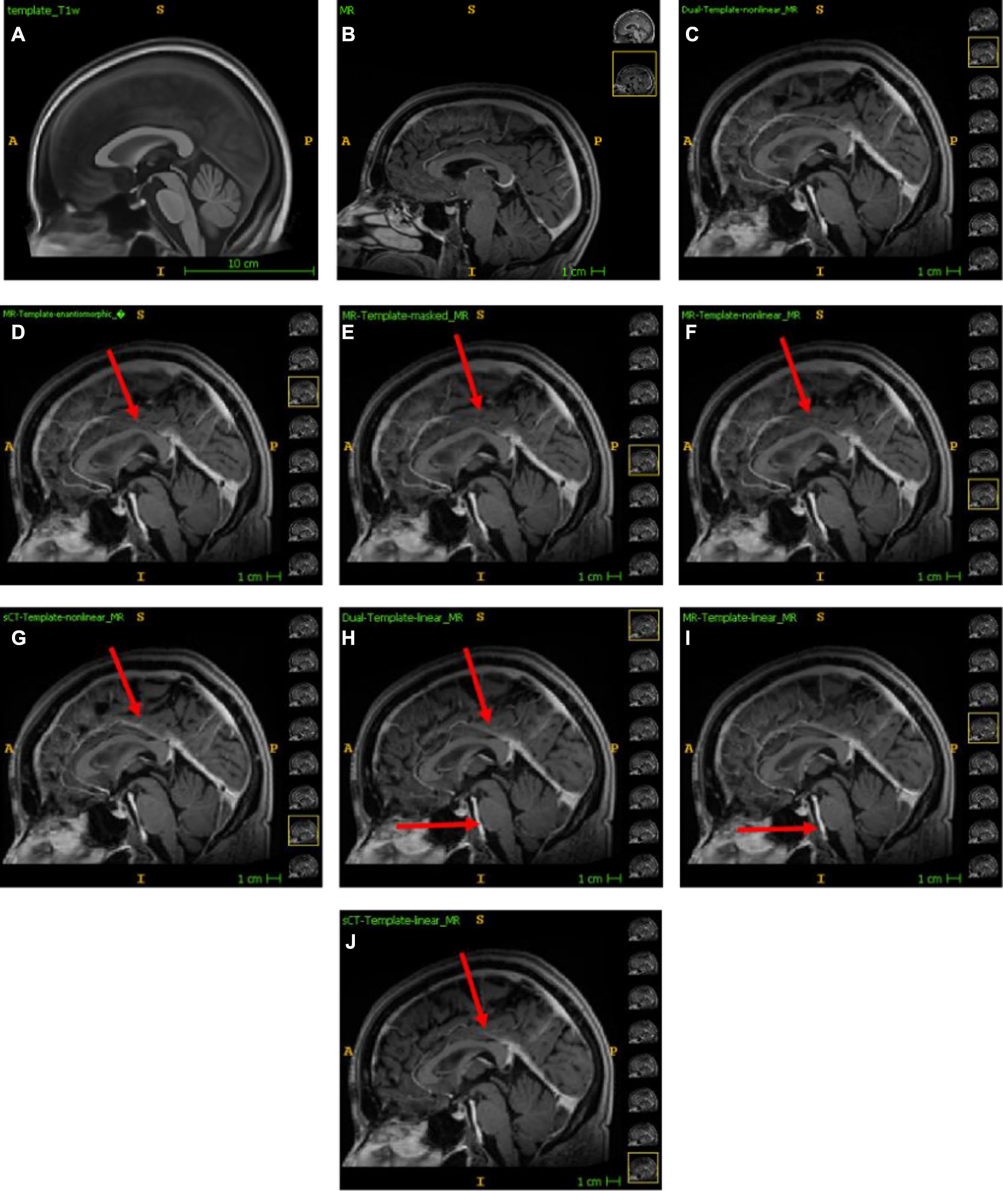
Comparison of sagittal slices of the MR images registered on to the template space **(A)**, the native space **(B)** using the Dual-Template-non-linear **(C)**, MR-Template-enantiomorphic **(D)**, MR-Template-masked **(E)**, MR-Template-non-linear **(F)**, sCT-Template-non-linear **(G)**, Dual-Template-linear **(H)**, MR-Template-linear **(I)**, and sCT-Template-linear schemes**(J)**. The images are shown from highest to lowest score, with the Dual-Template-non-linear scoring a 5 and the sCT-Template-linear scoring a 2. The red arrows point to distortions in the corpus callosum and brainstem.

Anatomical distortions were visible with the non-linear schemes across all templates, with the Dual-Template-non-linear scheme being the least affected. The parts of the brain that were most impacted were the midline, the corpus callosum, and the straight sinus. Furthermore, the shape of the skull was frequently distorted in all but the Dual-Template-non-linear scheme. An example scoring for participant 036 is shown in Figures 3, 4. For this participant, the Dual-Template-non-linear scored highest (5), with the MR-Template-enantiomorphic scoring a 4. The MR-Template-masked, MR- Template-non-linear, sCT-Template-non-linear, and Dual-Template-linear scored a 3. Finally, the sCT-Template-linear and MR-Template-linear scored a 2. More examples showing distortions can be found in the Supplementary material.

The masks created from the radiotherapy dose distributions worked well and, in most cases, the lesions in the final MR images were not visible. However, in the cases where lesions were sitting or crossing the midline, the enantiomorphic mask did not perform well. For those patients, the lesions were not masked appropriately and changes in the size, shape, and laterality of the tumours were observed. Examples are shown in the Supplementary materials. The MR-Template-enantiomorphic was the most inconsistent scheme, with some registrations scoring a 5 (highest), whilst other scoring a 1 (lowest).

The intra-rater reliability demonstrated good-to-excellent consistency with a single measures ICC of 0.806 (95% CI [0.777–0.883], *p* < 0.001).

### 3.2 Quantitative evaluation of registrations

**Figure 5:**
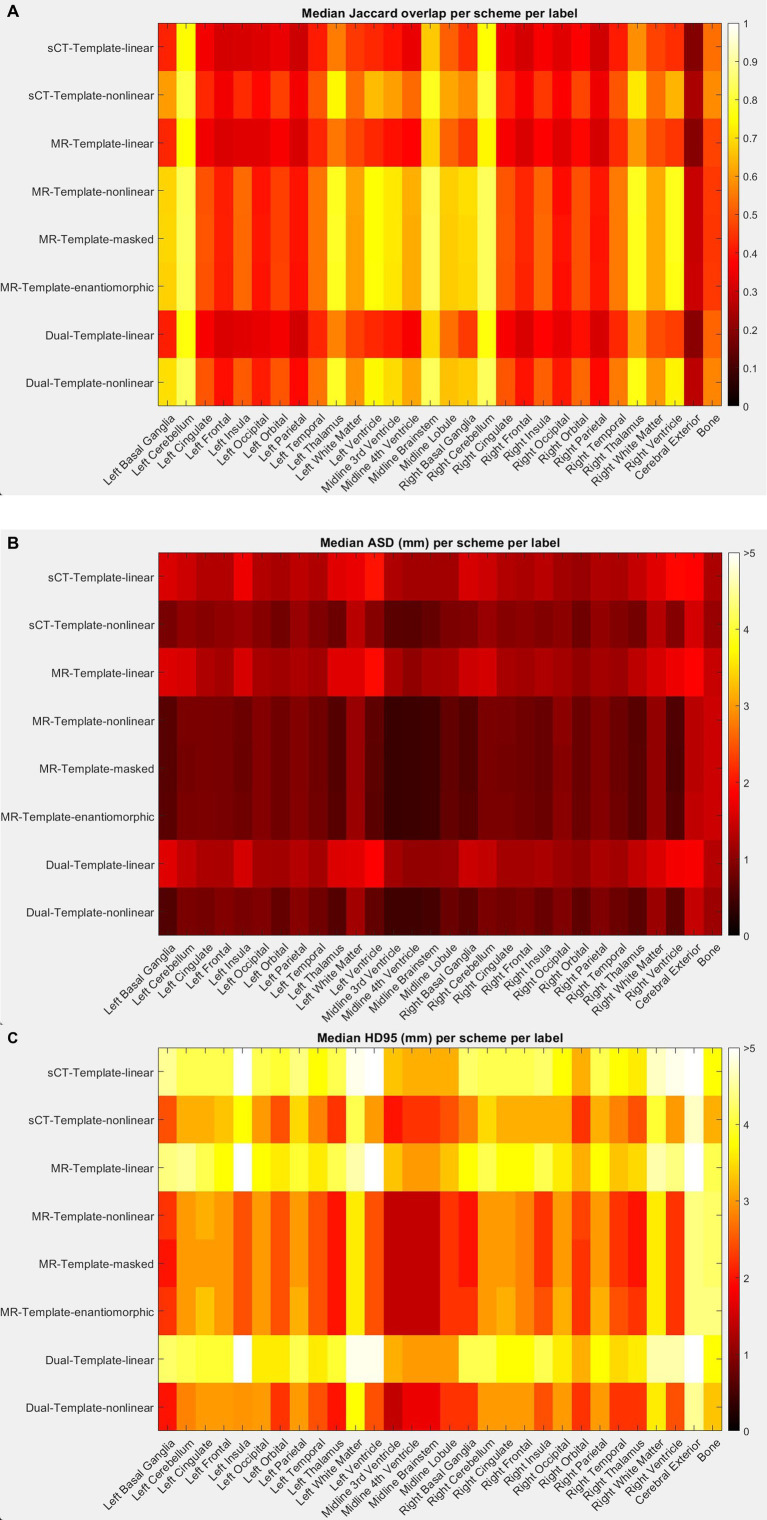
Median Jaccard **(A)**, median ASD **(B)**, and HD95 **(C)** scores for all participants across each registration scheme and for each structure.

Figure 5 shows the median Jaccard, ASD, and HD95 scores for each registration scheme for all participants. The values are depicted for the 30 structures. A pattern emerges: all the non-linear registrations perform similarly, on a population basis, across all labels apart from bone. For bone, sCT-Template and Dual-Template non-linear schemes performed better at each measure. Graphs with the median Jaccard, ASD, and HD95 scores for each scheme separately across all patients can be found in the Supplementary materials.

From Figure 5 it can be seen that, for the bone structure, the sCT-Template-non-linear and the Dual-Template-non-linear schemes provide the highest fidelity with lower (darker) ASD and HD95 and higher (lighter) Jaccard values. This is especially important in our population, as a large proportion has lesions, metastases, and meningiomas, located close to the skull.

In Table 2, the results are shown for the mean (SD) and median (IQR) for each registration scheme for Jaccard, ASD, and HD95. When reviewing the medians for each of the measures, the MR-Template-masked performed better across both Jaccard and ASD. The median Jaccard values for the MR-Template-non-linear and Dual-Template-non-linear schemes were very similar to that of the MR-Template-masked scheme, with slightly smaller IQRs. For HD95, the MR-Template-non-linear and MR-Template-masked performed similarly to the Dual-Template-non-linear.

**Table 2:** The mean, SD, median, and IQR for Jaccard, ASD, and HD95 across each registration scheme.

Registration method	Jaccard				ASD				HD95			
	Mean	SD	Median	IQR	Mean	SD	Median	IQR	Mean	SD	Median	IQR
‘sCT-Template-linear’	0.422	0.0416	0.398	0.063	1.503	0.2377	1.390	0.302	4.351	0.6508	4.123	0.730
‘sCT-Template-non-linear’	0.515	0.0449	0.503	0.052	1.077	0.2051	0.974	0.159	3.314	0.5595	3.000	0.248
‘MR-Template-linear’	0.425	0.0549	0.409	0.069	1.576	0.9657	1.338	0.281	4.516	2.5497	3.924	0.692
‘MR-Template-non-linear’	0.566	0.0555	0.552	0.052	1.010	0.8968	0.824	0.154	3.256	2.4149	**2.639**	**0.705**
‘MR-Template-masked’	**0.567**	**0.0557**	**0.555**	**0.055**	1.006	0.8960	**0.808**	**0.162**	3.254	2.4139	**2.639**	**0.716**
‘MR-Template-enantiomorphic’	0.550	0.0962	0.550	0.055	1.242	1.4063	0.815	0.218	3.877	3.7451	2.725	0.819
‘Dual-Template-linear’	0.434	0.0408	0.417	0.067	1.429	0.2146	1.314	0.268	4.147	0.5945	3.871	0.637
‘Dual-Template-non-linear’	0.564	0.0407	0.554	0.054	**0.918**	**0.1774**	0.829	0.129	**2.965**	**0.5392**	**2.639**	**0.657**

In bold are the highest values for Jaccard and the lowest values for ASD and HD95.

The Friedman test revealed no statistically significant differences between the medians of the Dual-Template-non-linear, the MR-Template-non-linear, and MR-Template enantiomorphic with Dunn-Bonferroni adjusted values of *p* = 1.000 for all pairwise comparisons across Jaccard, ASD, and HD95. When comparing between the medians of the Dual-Template-non-linear and the MR-Template-masked, the Dunn-Bonferroni adjusted values were p = 1.000 for Jaccard and HD95 and *p* = 0.931 for ASD. When comparing linear with non-linear registration methods, there was a statistically significant difference (*p* = <0.001) across all medians for all schemes across Jaccard, ASD, and HD95.

The MR-Template-enantiomorphic registration also showed the largest variation in the quantitative analysis, with the means across all measures having larger SDs than all other templates. For the ASD and HD95, the SDs for the MR-Template enantiomorphic were more than triple that of the Dual-Template ones. The IQRs for this template, which by their nature exclude the outliers, were similar to other templates.

The one-way repeated measures ANOVA for the means for Jaccard, ASD, and HD95 revealed no statistically significant differences between the Dual-Template-non-linear, the MR-Template-non-linear, and the MR-Template-masked, with Bonferroni-adjusted *p*-values of 1.000 for all pairwise comparisons between these templates. There was no statistically significant difference between the Dual-Template-non-linear and the MR-Template-enantiomorphic, with adjusted *p* = 0.953, *p* = 1.000, and *p* = 0.707 for ASD, Jaccard, and HD95, respectively. All linear registration methods were significantly different from their non-linear equivalents for the Jaccard. For ASD and HD95, they were not significantly different from the MR-Template-enantiomorphic. The latter showed non-significant differences (*p* > 0.05) to all the other registration methods for ASD and HD95.

The Dual-Template-non-linear scheme took an average of 202 s per patient to complete with the MR-Template-non-linear and the sCT-Template-non-linear taking 90 and 123 s, respectively.

**Figure 6:**
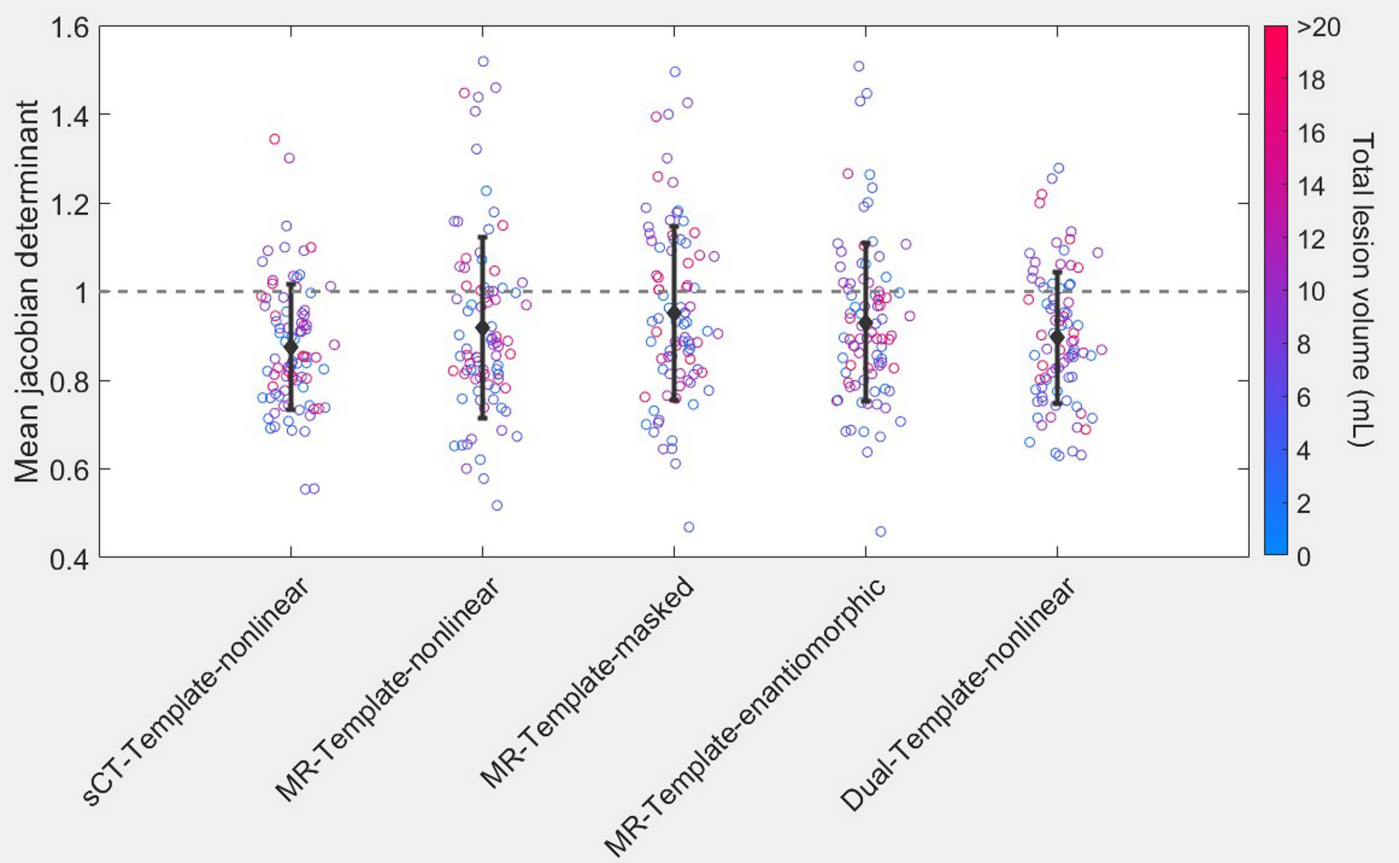
The mean Jacobian determinant values within the lesions shown across each non-linear scheme.

The in-lesion Jacobian determinant analysis revealed values mostly below 1 across all non-linear schemes. That signifies that, for the majority of the participants, the schemes aimed to contract down the lesion. All the values for each scheme are shown in Figure 6 and are colour coded according to total lesion volume. The MR-Template-masked performed the best, which is to be expected considering a masking technique was used to minimise changes within the lesion area. Despite that, the scheme on some occasions was inconsistent, with Jacobian determinant values of about 0.5 or 1.5 for some participants. Both schemes that utilised CT in the registration pipeline were more consistent, with the Jacobian determinant values varying around 1 less than the MR-only schemes. The Dual-Template-non-linear scheme had the smallest range of values across all participants.

In Table 3, the Jacobian determinant analysis with regards to negative values across the whole of the images is shown. The were no negative Jacobian values for the Dual-Template-non-linear and MR-Template enantiomorphic, only one participant for the MR-Template non-linear scheme, and two for the MR-Template masked. Finally, the sCT-Template-non-linear had six subjects with negative Jacobian values.

**Table 3:** The negative Jacobian determinant values across all non-linear registration schemes.

Registration scheme	Number of subjects	Number of voxels with negative Jacobian (% of total voxels)	Minimum Jacobian value
sCT-Template-non-linear	6	1,643 (0.09567%)	−0.367
		474 (0.02760%)	−0.211
		442 (0.02574%)	−0.185
		311 (0.01811%)	−0.177
		253 (0.01473%)	−0.161
		212 (0.01234%)	−0.145
MR-Template-non-linear	1	672 (0.03913%)	−0.124
MR-Template-masked	2	3 (0.00017%)	−0.005
		1 (0.00006%)	−0.001
MR-Template-enantiomorphic	0	—	—
Dual-Template-non-linear	0	—	—

## 4 Discussion

In this study, we qualitatively and quantitatively assessed registrations in 85 participants. To our knowledge, this is the largest study to date that has assessed different registration schemes using adult brain images containing tumours. The new registration method that we have proposed uses both MR and CT data to achieve optimal transformation of radiotherapy images and dose distributions into a common template space. Utilising the superior soft tissue contrast of MR in conjunction with the anatomical accuracy of CT, we postulated that, by using dual registration (Dual-Template scheme), images brought to a common template will combine the best of both worlds: a higher level of soft tissue contrast through MR images, whilst also retaining the correct bone and ventricle anatomy through CT.

When aiming to carry out group-level statistical analysis, the fidelity of the underlying anatomy is paramount. This is even more true when aiming to assess the damage caused by radiation treatment or associate damaged tissue with radiotherapy doses. In most cases, the underlying anatomy is only reviewed around the tumour, as the radiotherapy doses are delivered in a co-planar manner by modern radiotherapy techniques. However, in specialty cases such as SRS, where the radiotherapy doses are delivered in a non-coplanar way, the underlying fidelity of the anatomy of the whole brain including the skull is required. Bosma et al., in their recent review, recommended metrics for assessing deformable registration algorithms in the radiotherapy setting (Bosma et al., 2024). They advise the combination of quantitative and qualitative metrics (Bosma et al., 2024) as an extension of the metrics that were described initially in the AAPM TG-132 (Brock et al., 2017).

In our study we have used a subset of these metrics to ensure that our analysis is robust. Both qualitative and quantitative metrics strengthen the results of our study, with overlap, distance-to-agreement, and boundary-based evaluation metrics. Non-linear registrations pose a risk, amongst other problems, of collapsing or overexpanding voxels, which can adversely affect dose deformation, accumulation, or any subsequent dose calculations using the deformed images (Crum et al., 2004; Razdevsek et al., 2022; Hussein et al., 2021; Schultheiss et al., 2012; Zhang et al., 2018). Our evaluation of the Jacobian determinant values has shown that the Dual-Template scheme is not affected by such issues. Additionally, the Jacobian determinant values within the lesions showed that, even without the use of masks, the Dual-Template-non-linear scheme performed consistently across all lesion volumes for all participants.

CT images lack soft tissue contrast, which can adversely affect registrations. Because of this, the presence of tumours or oedema will not affect CT-based registrations to the same degree as MR-based registrations. Qualitatively, the sCT-Template-non-linear registration scheme was the most consistent, with the majority of registrations scoring 2 or 3, although it still performed overall poorly. Quantitatively, it provided the best fidelity in the bone structure along with the Dual-Template-non-linear scheme. Despite that, the scheme was still affected by the standard issues that burden non-linear registrations (see Supplementary materials for details).

In the qualitative assessment, the Dual-Template-non-linear was the clear favourite, with 60 participants scoring 4 and 5. The qualitative assessment provided new insights that the quantitative measures alone would have been missed, including distortions in the skull and elongation of the brain. Using the proposed Dual-Template to bring images on to a template space minimises these distortions, as it uses both MR and CT data. Additionally, it does not require the creation of masks, which requires highly skilled professionals and is very time consuming.

The issues that arise with using non-linear registrations have been documented widely (Hub et al., 2009; Brett et al., 2001; Sdika and Pelletier, 2009). Geometric distortions (of the skull, lesion, internal anatomy, and incorrect displacement of doses) along with a lack of validation techniques are a few of the many reasons why non-linear transformation techniques are not commonly used in radiotherapy practise (Crum et al., 2004; Hussein et al., 2021; Schultheiss et al., 2012; Brock et al., 2017). Jühling et al. (2024), postulated that, for lesioned brains where VLSM analysis is required, affine registrations alone are sufficient. In our study, we have shown that this is not the case, with linear registrations of all schemes performing poorly. When aiming to perform subject to template registrations, as required for voxel-wise analysis, non-linear registrations are necessary (Monti et al., 2020; Mcwilliam et al., 2023; Palma et al., 2020; Hellier et al., 2002). The use of the Dual-Template can aid in minimising distortions (see Supplementary material for examples). The measures used for the quantitative assessment of the registration schemes provided a well-balanced approach, as they included both overlap measures (Jaccard) and distance measures (ASD and HD95). In this way, we have evaluated not only the overlap but also whether the boundaries of the segmented structures are accurately placed.

Different registration algorithms can produce varying results and, occasionally, introduce artefacts (Ashburner and Friston, 2000; Mechelli et al., 2005). Monti et al. (2018), Monti et al. (2020) suggest the use of a non-linear scheme (*Β*-spline algorithm) and a masking approach. Palma et al. (2020) recommend two algorithms (B-spline or Demons) but point out that both have advantages and disadvantages (Hub et al., 2009). Distortions were visible in all the non-linear schemes, with only the Dual-Template non-linear scheme not being adversely affected.

Overall, non-linear registrations outperformed their linear counterparts, with the proposed Dual-Template-non-linear registration scheme being the most consistent, showing the smallest SDs and smallest IQRs across all three measures. It was also statistically non-inferior compared to already widely used existing methods (Monti et al., 2018; Monti et al., 2020; Mcwilliam et al., 2023; Palma et al., 2020; Wilson et al., 2024; Wilson et al., 2022; Aznar et al., 2026). Additionally, CT-only based schemes performed worse than other schemes, both qualitatively and quantitatively. The addition of MR with the use of the Dual-template non-linear scheme substantially improved the alignment, with non-linear MR-only registrations also producing good but less consistent results. In radiotherapy, the majority of the patients have a CT scan available. Consequently, using CT data to improve registration fidelity may help to improve the outcomes of voxel-wise analyses.

Distortions in the skull are not normally picked up by quantitative measures as they are based on segmentations that focus on the brain and involve a brain extraction algorithm (skull-stripping), at the early stages of processing. In our study, we created a bone structure that outlined the skull. All the MR-Template schemes performed worse than the sCT-Template and Dual-Template schemes for this structure. This is to be expected, as the bone-soft tissue contrast is better in CT than MR imaging.

Care should be taken when using masking techniques, such as enantiomorphic used by Jühling et al. (2024). These can be very useful in instances where the lesions are not close to the midline. However, for lesions that are located on or crossing the midline, the masking can inadvertently change the shape, size, and location of the original tumour. In our study, the MR-Template-enantiomorphic registration also showed the largest variation in both the quantitative and qualitative assessment. Additionally, there was no statistically significant difference between the MR-Template-enantiomorphic and any of the other registration methods when comparing the means across ASD and HD95, which further reinforces the need for using multiple metrics to assess performance and is suggestive of the inconsistency of the method.

It has been shown that the use of age-specific reference data improves the alignment of the segmented structures, and this is what we saw in our case (Ashburner and Friston, 2000; Fillmore et al., 2015; Good et al., 2001; Thompson et al., 2001; Huang et al., 2010). Ashburner and Friston (2000), amongst others, have documented the various issues that arise from using reference data from young adults instead of age-appropriate reference data. Our choice to use reference data from Rorden et al. (2012) and not a template based on young reference data has likely aided registration into template space (Monti et al., 2020). Other studies using voxel-based analyses (VBA) in radiotherapy use templates that have been derived from the patient population (Wilson et al., 2022). We believe that choosing published reference data increases the generalisability of the results but are aware that it can negatively impact the quality of the registrations.

Equal weightings for the CT and MR similarity terms were applied, with other weighting combinations not fully explored. The impact of using different weightings on the different registration methods warrants further investigation in future work.

Our choice of the radiotherapy dose distributions instead of manually drawn masks for the MR-template-masked and MR-template-enantiomorphic registrations is a novel way to delineate the lesions. The radiotherapy dose distributions are a more robust way of delineation, as these are not user dependant, thereby reducing intra- and inter-user variability. Finally, the creation of masks can be easily automated where dosimetric data from radiotherapy plans are available.

For the creation of the masks, we selected a dose level of 15 Gy as the cut-off point. This meant that in the cases where the dose prescription was higher than 15 Gy, a circular rim of healthy brain was omitted from the registration. For two participants, one with a dose prescription of 14 Gy and one with a prescription of 12 Gy, the opposite occurred, i.e., lesioned brain areas had not been masked out. Considering the sharp dose-drop-off of SRS treatments, we estimated that the healthy brain that would be replaced by the 15 Gy mask for a 1 cc lesion receiving 22 Gy would be 1.7 cc. Therefore, we do not believe that our choice of cut-off had a significant impact on the quality of the registrations.

A limitation of our study was that the qualitative review of the registrations was carried out by one reviewer. Despite the fact that they were experienced and the intra-rater reliability was proved, there could have been observer bias as they were not blinded to the registration method they were reviewing. Another important drawback of our study is that it was performed at a single centre. However, Nottingham University Hospitals NHS Trust is a regional provider of SRS and, consequently, the participants covered the area of the East Midlands (~10% of the English population). Patients were scanned in MRI scanners from all three manufacturers and with field strengths of both 1.5 and 3 T. The population included some meningioma patients who had prior surgery. Patients with oedema were also included in the population, but the impact of the above on the performance of the different registration methods was not assessed. This could potentially limit the generalisability of the results to other patient populations.

## 5 Conclusion

The Dual-Template-non-linear scheme was qualitatively superior. Quantitatively, the scheme was non-inferior to the MR-Template-masked scheme. The addition of CT to the MR-Template provided higher fidelity in areas such as the skull and minimised non-linear distortions within the brain. When aiming to carry out group-level statistical analyses in populations with brain lesions, and especially in radiotherapy, we propose the use of Dual-Template-non-linear registration as the most robust way of bringing participant data on to a common template space.

## Data Availability

The defaced image sets, along with the raw numerical data for the analysis and the scripts have been shared in a publicly available depository, available at: https://doi.org/10.17605/OSF.IO/5UPQ8.

## Footnotes

1 1 Gy = 1 Joule/kilogram (J/kg) as per ICRU 93(26).
