# Supplementary material for: Comparison of subject-to-template registration schemes using CT and MR radiotherapy images with brain lesions
Source: Front Neurosci. 2026 Jul 28;20:1858490. doi: 10.3389/fnins.2026.1858490 (PMC13457462; doi:10.3389/fnins.2026.1858490)
Supplement: Supplementary file 1 [file Supplementary_file_1.docx]

## S1: Inclusion and exclusion criteria

In Table S1 below the inclusion and exclusion criteria for both cohorts of the study are shown.

Table S1: Inclusion and exclusion criteria for the prospective and retrospective cohorts of the CoDe-B-Rad study.

|  | Inclusion criteria | Exclusion criteria |
| --- | --- | --- |
| Prospective cohort | Age: above 18 years  Diagnosis of brain metastases or meningioma, where the treatment is going to be SRS  Karnofsky Performance Status (KPS) ≥70  Established diagnosis of cancer with absent or controllable primary disease  Tumour volume of less than 20cc  Life expectancy >6 months  Able to give informed consent | Previous RT to the brain, including SRS  Previous surgery to the brain  Not willing or not able to give informed consent |
| Retrospective cohort | Age: above 18 years  Diagnosis of Meningioma  Most recent MRI scan (within 1 year) that shows stable appearances.  SRS treatment at least 1 year prior to recruitment | Previous radiotherapy other than SRS  Unwilling or unable to give written informed consent. |

## S2: Structure combinations

Table S2 below shows the structure combinations to condense the 138 MALP-EM structures down to 28 structures.

Table S2: The 138 cortical and subcortical MALP-EM structures combined in 28 structures used for the quantitative analysis.

| **Label** | **Region** | **MALP-EM substructures** |
| --- | --- | --- |
| 1 | Left Basal Ganglia | Left Accumbens Area, Left Caudate, Left Pallidum, Left Putamen |
| 2 | Left Cerebellum | Left Cerebellar Exterior, Left Cerebellum White Matter |
| 3 | Left Cingulate | Left Anterior Cingulate Gyrus, Left Middle Cingulate Gyrus, Left Posterior Cingulate Gyrus, Left Subcallosal Area |
| 4 | Left Frontal | Left Basal Forebrain, Left Frontal Pole, Left Gyrus Rectus, Left Medial Frontal Cortex, Left Middle Frontal Gyrus Medial Segment, Left Precentral Gyrus Medial Segment, Left Superior Frontal Gyrus Medial Segment, Left Opercular Part of the Inferior Frontal Gyrus, Left Orbital Part of the Inferior Frontal Gyrus, Left Posterior Part of the Inferior Frontal Gyrus |
| 5 | Left Insula | Left Anterior Insula, Left Central Operculum, Left Frontal Operculum |
| 6 | Left Occipital | Left Calcarine Cortex, Left Cuneus, Left Inferior Occipital Gyrus, Left Middle Occipital Gyrus, Left Superior Occipital Gyrus, Left Occipital Fusiform Gyrus, Left Occipital Pole |
| 7 | Left Orbital | Left Anterior Orbital Gyrus, Left Lateral Orbital Gyrus, Left Posterior Orbital Gyrus |
| 8 | Left Parietal | Left Angular Gyrus, Left Precuneus, Left Supramarginal Gyrus, Left Superior Parietal Lobule, Left Postcentral Gyrus Medial Segment, Left Postcentral Gyrus |
| 9 | Left Temporal | Left Fusiform Gyrus, Left Entorhinal Area, Left Hippocampus, Left Inferior Temporal Gyrus, Left Middle Temporal Gyrus, Left Parahippocampal Gyrus, Left Planum Polare, Left Planum Temporale, Left Superior Temporal Gyrus, Left Temporal Pole, Left Transverse Temporal Gyrus |
| 10 | Left Thalamus | Left Thalamus Proper |
| 11 | Left White Matter | Left Cerebral White Matter |
| 12 | LT Ventricle | Left Inferior Lateral Ventricle, Left Lateral Ventricle |
| 13 | Midline Brainstem | Brain Stem |
| 14 | Midline Lobule | Cerebellar Vermal Lobules I–V, Cerebellar Vermal Lobules VI–VII, Cerebellar Vermal Lobules VIII–X |
| 15 | Right Basal Ganglia | Right Accumbens Area, Right Caudate, Right Pallidum, Right Putamen |
| 16 | Right Cerebellum | Right Cerebellar Exterior, Right Cerebellum White Matter |
| 17 | Right Cingulate | Right Anterior Cingulate Gyrus, Right Middle Cingulate Gyrus, Right Posterior Cingulate Gyrus, Right Subcallosal Area |
| 18 | Right Frontal | Right Basal Forebrain, Right Supplementary Motor Cortex, Right Frontal Pole, Right Gyrus Rectus, Right Medial Frontal Cortex, Right Middle Frontal Gyrus Medial Segment, Right Precentral Gyrus Medial Segment, Right Superior Frontal Gyrus Medial Segment, Right Opercular Part of the Inferior Frontal Gyrus, Right Orbital Part of the Inferior Frontal Gyrus, Right Posterior Part of the Inferior Frontal Gyrus |
| 19 | Right Insula | Right Anterior Insula, Right Posterior Insula, Right Central Operculum, Right Frontal Operculum |
| 20 | Right Occipital | Right Calcarine Cortex, Right Cuneus, Right Inferior Occipital Gyrus, Right Middle Occipital Gyrus, Right Superior Occipital Gyrus, Right Occipital Fusiform Gyrus, Right Occipital Pole |
| 21 | Right Orbital | Right Anterior Orbital Gyrus, Right Lateral Orbital Gyrus, Right Posterior Orbital Gyrus |
| 22 | Right Parietal | Right Angular Gyrus, Right Precuneus, Right Supramarginal Gyrus, Right Superior Parietal Lobule, Right Postcentral Gyrus Medial Segment, Right Postcentral Gyrus |
| 23 | Right Temporal | Right Fusiform Gyrus, Right Entorhinal Area, Right Hippocampus, Right Inferior Temporal Gyrus, Right Middle Temporal Gyrus, Right Parahippocampal Gyrus, Right Planum Polare, Right Planum Temporale, Right Superior Temporal Gyrus, Right Temporal Pole, Right Transverse Temporal Gyrus |
| 24 | Right Thalamus | Right Thalamus Proper |
| 25 | Right White Matter | Right Cerebral White Matter |
| 26 | Unknown | Cerebrospinal Fluid, Left Cerebral Exterior, Left Vessel, Right Cerebral Exterior, Right Vessel, Fourth Ventricle, Optic Chiasm |
| 27 | Rt Ventricle | Right Inferior Lateral Ventricle, Right Lateral Ventricle |
| 28 | Left Basal Ganglia | Left Accumbens Area, Left Caudate, Left Pallidum, Left Putamen |

## S3: Jaccard results for all patients for each individual registration scheme

The Jaccard, ASD and HD95 results for each registration scheme are shown in Figure S1, Figure S2 and Figure S3 respectively. Each participant represents the same column across each scheme. The colour scheme for each measure is depicted at the side of each graph.

Figure S1: Jaccard results for all participants separated by scheme.

Figure S2: ASD results for all participants separated by scheme.

Figure S3: HD95 results for all participants separated by scheme.

## S4: Examples of geometric and dosimetric distortions

In the images below (Figure S4) examples of geometric distortions can be seen for the non-linear registration schemes for different participants. These were more pronounced in the sCT-Template schemes rather than the schemes that utilised MR images (alone or in combination with CT).

Figure S4: The sCT-Template-non-linear registrations for participants 028 (left), 056 (middle) and 072 (right). The distortions introduced by the non-linear registration have been highlighted with red arrows.

The images in Figure S5 show an example where the MR-Template-masked has resulted in elongation of the brain and distortion of the shape of the skull.

Figure S5: Registrations for participant 022 with the Dual-Template-non-linear registration (left) and the MR-Template-masked registration (right). The elongation at the superior end of the brain and skull is clear in the right image.

In Figure S6 distortions for participant 023 are more pronounced with the MR-Template-masked (top) than the Dual-Template-non-linear registration (bottom). These distortions are the visible on the midline and in the superior part of the dura.

Figure S6: (top) MR-Template-masked registration for participant 023. (bottom) The Dual-Template-non-linear registration for the same participant. The distortions are not as pronounced as with the MR-Template-masked.

In Figure S7 the dose distribution of the radiotherapy plan has been super-imposed on the images for participant 028 after they have been brought into template space. It is clear that the dose has been distorted by the non-linear transformation of the MR image in template space.

Figure S7: The radiotherapy dose overlayed on the transformed MR image. The distortions introduced by the transformation clearly affect the dose distribution.

In Figure S8, the lesion for participant 076 was sat on the midline, and the enantiomorphic mask displaced it from the left hemisphere to the right, also significantly changing the size and shape.

Figure S8: (top) MR-Template-enantiomorphic has displace the location of the tumour from the left hemisphere to the right. (bottom) The Dual-Template-non-linear with the true location of the tumour. The red circles enclose the tumour.
